# Modulation of defensive reflex conditioning in snails by serotonin

**DOI:** 10.3389/fnbeh.2015.00279

**Published:** 2015-10-23

**Authors:** Vyatcheslav V. Andrianov, Tatiana K. Bogodvid, Irina B. Deryabina, Aleksandra N. Golovchenko, Lyudmila N. Muranova, Roza R. Tagirova, Aliya K. Vinarskaya, Khalil L. Gainutdinov

**Affiliations:** ^1^Laboratory of Neurobiology, Institute of Fundamental Medicine and Biology, Kazan Federal UniversityKazan, Russia; ^2^Group of Biophysics, Zavoisky Physical-Technical Institute, Russian Academy of SciencesKazan, Russia; ^3^Department of Biomedical Sciences, Volga Region State Academy of Physical Culture, Sport and TourismKazan, Russia; ^4^Laboratory of Cellular Neurobiology of Learning, Institute of High Nerve Activity and Neurophysiology, Russian Academy of SciencesMoscow, Russia

**Keywords:** serotonin, associative learning, identified neurons, membrane potential, threshold potential, snail

## Abstract

**Highlights**

- Daily injection of serotonin before a training session accelerated defensive reflex conditioning in snails.
- Daily injection of 5-hydroxytryptophan before a training session in snails with a deficiency of serotonin induced by the “neurotoxic” analog of serotonin 5,7-dihydroxytryptamine, restored the ability of snails to learn.
- After injection of the “neurotoxic” analogs of serotonin 5,6- and 5,7-dihydroxytryptamine as well as serotonin, depolarization of the membrane and decrease of the threshold potential of premotor interneurons was observed.

We studied the role of serotonin in the mechanisms of learning in terrestrial snails. To produce a serotonin deficit, the “neurotoxic” analogs of serotonin, 5,6- or 5,7-dihydroxytryptamine (5,6/5,7-DHT) were used. Injection of 5,6/5,7-DHT was found to disrupt defensive reflex conditioning. Within 2 weeks of neurotoxin application, the ability to learn had recovered. Daily injection of serotonin before a training session accelerated defensive reflex conditioning and daily injections of 5-HTP in snails with a deficiency of serotonin induced by 5,7-DHT restored the snail's ability to learn. We discovered that injections of the neurotoxins 5,6/5,7-DHT as well as serotonin, caused a decrease in the resting and threshold potentials of the premotor interneurons LPa3 and RPa3.

## Introduction

One of the widespread and well-investigated transmitters in the nervous system is serotonin (Kandel and Schwartz, 1982; Sakharov, 1990; Crow, 2004; Gillette, 2006). Within a short period of time serotonin (5-HT) was identified as a neurotransmitter in both mollusks and mammals (Whitaker-Azmitia, 1999; Marinesco et al., 2004; Lee et al., 2009). The serotoninergic system plays an important role in the modulation of stress-induced excitability (arousal) and defensive behavior (Il-Han et al., 2010). The “5-HT neurons dispersed throughout the CNS of lophotrochozoan invertebrates (mollusks and leeches) are analogous to vertebrate 5-HT neurons concentrated in the raphe nuclei of the mid- and hindbrain: they innervate specific central pattern generators and other circuits of the CNS, receive feedback from them, and support general behavioral arousal” (Gillette, 2006). It has been shown that long-term facilitation in connections between sensory and motor neurons of the gill withdrawal reflex is mediated by 5-HT and this form of synaptic plasticity was found to be a critical cellular mechanism in behavioral sensitization (Barbas et al., 2003; Hawkins et al., 2006; Hart et al., 2011). In connection to the discovery of the relationship between the level of 5-HT in the hemolymph of mollusks and the sensitization of reflexes (Levenson et al., 1999; Hernadi et al., 2008), a lot of experiments have been conducted using manipulation of the 5-HT system to investigate cellular analogs of learning (Lent and Dickinson, 1984; Gadotti et al., 1986; Glanzman and Krasne, 1986; Jahan-Parwar et al., 1987; Vehosvzky et al., 1989; Mauelshagen et al., 1996; Kemenes, 1997; Malyshev et al., 1997; Shevelkin et al., 1997; Gainutdinov et al., 1999; Balaban, 2002; Burrell and Sahley, 2005; Jing et al., 2009). It was found that the injection of the neurotoxin 5,7-DHT led to a significant decrease in the withdrawal reflex caused by tail shock and inhibited the heterosynaptic facilitation between the sensory neuron and the subsequent cells in *Aplysia* (Glanzman et al., 1989). Balaban et al. (1987) showed that the pairing of food presentation and electrical stimulation didn't result in changes in responses to food in 5,7-DHT-injected snails, whereas in vehicle-injected snails defensive reactions were observed. Furthermore, injection of 5,7-DHT led to the disruption of long-term sensitization in *Aplysia* (Glanzman et al., 1989) and in snails (Balaban and Bravarenko, 1993; Gainutdinov et al., 1999). At the same time, it has been shown that although the intensity of the conditioning strongly decreases in leeches after depletion of serotonin by 5,7-DHT, they didn't lose the ability to learn (Burrell and Sahley, 1999). Thus, a considerable wealth of experimental material has been accumulated which points to the association between the functioning of the 5-HT- system of mollusks and their ability to learn (in behavioral experiments). However, the questions regarding to the specific mechanisms (and/or pathways) of 5-HT participation in associative learning and the role of specific neurons in these processes remain open. These findings and questions motivated us to investigate the role of 5-HT in the mechanisms of learning by behavioral and electrophysiological methods, using the “neurotoxic” analogs of serotonin 5,6/5,7-DHT, and the precursor of 5-HT syntheses, 5-hydroxytryptophan (5-HTP).

## Materials and methods

### Experimental animals

For the experiments, the terrestrial snails *Helix lucorum* from the Crimean population, were used. The nervous system of these snails has been well described (Schmalz, 1914; Kilias, 1985; Balaban, 1993).

All experimental procedures are in compliance with the Guide for the Care and Use of Laboratory Animals published by the National Institutes of Health and Directive 2010/63/EU of the European Parliament and of the Council of 22 September 2010. Snails were stored asleep. Prior to the experiments, the snails were kept for no less than 2 weeks in a glass terrarium in a humid atmosphere at room temperature (each group in a separate terrarium) (Article 33 of Directive 2010/63/EU). All groups were housed in separate terrariums which were kept together all the time in the same room under the same conditions. The electrophysiological measurements were carried out in isolated preparations the day after training. Prior to the preparation procedure, snails were anesthetized (Article 14 of Directive 2010/63/EU) by 30 min of immersion in water mixed with ice.

### Drugs

#### 5,6/5,7-DHT

In the experiments the “neurotoxic” analogs of serotonin 5,6- and 5,7-dihydroxytryptamine (5,6/5,7-DHT) were used. Their effects on defensive reflex conditioning of snails and the electrical characteristics of premotor interneurons were investigated. They selectively destroy the 5-HT elements in the nervous system, particularly in nerve terminals, thus decreasing the level of 5-HT (Gadotti et al., 1986; Glanzman and Krasne, 1986; Hernádi et al., 1992). 5,6-DHT (Sigma) was injected into snails twice at doses of 15 mg/kg with an interval of 7 days for a total dose of 30 mg/kg. 5,7-DHT (Sigma) was injected into snails once at a dose of 20 mg/kg (Balaban et al., 1987). The neurotoxins were dissolved in 0.1 ml of saline solution (SS). In addition, ascorbic acid was added to the solution as an antioxidant to achieve a concentration of 0.1%. The injection of 0.1 ml of SS (with the addition of ascorbic acid to achieve a concentration of 0.1%) was used as a control. The injection of only 5,6-DHT without training served as an alternative control. One month after the injection of 5,6/5,7-DHT the 5-HT-containing neurons were selectively labeled with brown pigmentation, pointing to capture of 5,6/5,7-DHT by these cells. This phenomenon has been very well described earlier (Glanzman and Krasne, 1986; Balaban et al., 1987; Vehosvzky et al., 1989). Therefore, we did not take photos of the off-labeled cells.

#### 5-HT

In another series of experiments the influence of serotonin (5-HT) on defensive reflex conditioning in snails and the electrical characteristics of premotor interneurons was investigated. 5-HT (Sigma) was injected into snails daily 1 h before a training session at a dose of 10 mg/kg. 5-HT was dissolved in 0.1 ml of SS, in addition ascorbic acid was added to the solution as an antioxidant to achieve a concentration of 0.1%. The injection of 0.1 ml of SS (with the addition of ascorbic acid to achieve a concentration of 0.1%) was used as a control.

#### 5-HTP

In a third series of experiments the influence of the precursor to 5-HT synthesis 5-hydroxytryptophan (5-HTP) on defensive reflex conditioning in snails and the electrical characteristics of premotor interneurons was investigated. 5-HTP (Sigma) was injected into snails daily 1 h before a training session at a dose of 10 mg/kg. 5-HTP was dissolved in 0.1 ml of SS, in addition ascorbic acid was added to the solution as an antioxidant to achieve a concentration of 0.1%. The injection of 0.1 ml of SS (with the addition of ascorbic acid to achieve a concentration of 0.1%) was used as a control.

### Experimental groups

In the experiments we used the following groups of animals:

#### Group 1: Defensive reflex conditioning

Snails were trained to execute the defensive reflex on tapping on the shell (Balaban, 1993; Gainutdinova et al., 2003). Tapping on the shell (2 times) was used as a conditioned stimulus, which under normal conditions doesn't produce any defensive reaction in a snail. As an unconditioned stimulus a puff of air into the lung cavity orifice (pneumostome) was used, which produces the defensive reaction of pneumostome closure in animals. Only a complete closure of the pneumostome was taken as a “positive reaction” to the stimulus. Combinations of stimuli were presented with a random interval that ranged from 2 to 4 min (to prevent the elaboration of a conditioned reflex to time). The defensive reflex was trained according to 2 protocols:

1. snails which were trained to execute the defensive conditioned reflex on tapping on the shell according to the first protocol (*n* = 19). Defensive conditioned reflex elaboration was developed over a 3 day period as a result of presentation of 150–170 pairs of the conditioned and unconditioned stimuli: this protocol consisted of 2 daily sessions each of them consisting of 30 combinations. The conditioned stimulus was presented by double tapping on the shell with 1.5 g force;
2. snails which were trained to execute the defensive conditioned reflex on tapping on the shell according to the second protocol (*n* = 10). Defensive reflex elaboration was developed over a 6–7 day period as a result of the presentation of 300–350 combinations of the conditioned and unconditioned stimuli: this protocol consisted of 2 daily sessions of 30 and 15 combinations where the conditioned stimulus was presented by double tapping on the shell with 1.0 g force;
3. snails of active control for the defensive conditioned reflex which received identical presentation of the conditioned and unconditioned stimuli, but in an unpaired combination (*n* = 18).

#### Group 2: Effects of 5-HT and 5-HT neurotoxins on defensive reflex conditioning

1. snails which were trained to execute the defensive conditioned reflex on tapping on the shell according to the first protocol after injection of 5-HT (*n* = 7), snails were injected daily 1 h before a training session with 5-HT at a dose of 10 mg/kg dissolved in 0.1 ml of SS;
2. snails which were injected with 5-HT, but not trained (*n* = 6), snails were injected daily with 5-HT at a dose of 10 mg/kg dissolved in 0.1 ml of SS;
3. snails which were trained to execute the defensive conditioned reflex on tapping on the shell according to the first protocol after injection of 0.1 ml of SS (*n* = 16), the injection of SS was used as a control;
4. snails which were trained to execute the defensive conditioned reflex on tapping on the shell according to the first protocol on the next day after the second injection of “neurotoxic” analog of serotonin 5,6-DHT (*n* = 8), and 1 week after the second injection of 5,6-DHT (*n* = 8), 5,6-DHT (Sigma) was injected into snails twice at doses of 15 mg/kg with an interval of 7 days for a total dose of 30 mg/kg. 5,6-DHT was dissolved in 0.1 ml of SS;
5. snails which were injected by “neurotoxic” analog of serotonin 5,6-DHT, but not trained (*n* = 26), 5,6-DHT (Sigma) was injected into snails twice at doses of 15 mg/kg with an interval of 7 days for a total dose of 30 mg/kg. 5,6-DHT was dissolved in 0.1 ml of SS.

#### Group 3: Common effects of 5-HTP and 5,7-DHT on defensive reflex conditioning

1. snails which were trained to execute the defensive conditioned reflex on tapping on the shell according to the second protocol after the daily injection of the precursor of 5-HT syntheses, 5-HTP (*n* = 8), snails were injected daily 1 h before a training session with 5-HTP at a dose of 10 mg/kg dissolved in 0.1 ml of SS;
2. snails which were trained to execute the defensive conditioned reflex on tapping on the shell according to the second protocol after injection of SS in volume 0.1 ml (*n* = 12), the injection of SS was used as a control;
3. snails which were trained to execute the defensive conditioned reflex on tapping on the shell according to the second protocol on the next day after the injection of 5,7-DHT (*n* = 8), 5,7-DHT (Sigma) was injected into snails once at a dose of 20 mg/kg. 5,7-DHT was dissolved in 0.1 ml of SS;
4. snails which were trained to execute the defensive conditioned reflex on tapping on the shell according to the second protocol after the daily injection of 5-HTP before each training session to animals which had previously been injected by “neurotoxic” analogs of serotonin 5,7-DHT (*n* = 10). 5,7-DHT (Sigma) was injected into snails once 5 days before the training session at a dose of 20 mg/kg, snails were injected also daily 1 h before a training session with 5-HTP at a dose of 10 mg/kg. 5,7-DHT and 5-HTP were dissolved in 0.1 ml of SS.

#### Group 4: Effects of 5-HT and 5-HT- neurotoxins on the electrical characteristics of premotor interneurons in snails after defensive reflex conditioning

1. registration of the electrical characteristics of premotor interneurons in snails which were trained to execute the defensive conditioned reflex on tapping on the shell according to the first protocol after injection of 5-HT (*n* = 7), snails were injected daily 1 h before a training session with 5-HT at a dose of 10 mg/kg in 0.1 ml of SS;
2. registration of electrical characteristics of premotor interneurons of snails which were injected with 5-HT, but not trained (*n* = 6), snails were injected daily with 5-HT at a dose of 10 mg/kg in 0.1 ml of SS;
3. registration of the electrical characteristics of premotor interneurons in snails which were trained to execute the defensive conditioned reflex on tapping on the shell according to the first protocol on the next day after the second injection of 5,6-DHT (*n* = 8), and 1 week after the second injection of 5,6-DHT (*n* = 8), 5,6-DHT (Sigma) was injected into snails twice at doses of 15 mg/kg with an interval of 7 days for a total dose of 30 mg/kg. 5,6-DHT was dissolved in 0.1 ml of SS;
4. registration of the electrical characteristics of premotor interneurons in snails which were injected by 5,6-DHT, but not trained (*n* = 26), 5,6-DHT (Sigma) was injected into snails twice at doses of 15 mg/kg with an interval of 7 days for a total dose of 30 mg/kg. The neurotoxin was dissolved in 0.1 ml of SS;
5. registration of the electrical characteristics of premotor interneurons in snails which were trained to execute the defensive conditioned reflex on tapping on the shell according to the first protocol (*n* = 19).

### Intracellular recording

The nervous ring was immersed in a saline solution of the following composition: NaCl—80 mM, KCl—4 mM, CaCl_2_—10 mM, MgCl_2_—6 mM, NaHCO_3_—5 mM (or Tris—5 mM), pH—7.6–7.8. The electrical characteristics of the withdrawal interneurons of the snail's pneumostome closure reflex LPa3 and RPa3 (Balaban, 1993, 2002) were analyzed. The recordings of the electrical characteristics were carried out on the day after training. The measurements were conducted at room temperature (18–21°C) using intracellular glass microelectrodes with a resistance 10–30 MOm filled with 2.5 M KCl. The following parameters of the nervous cells were studied: resting membrane potential (the initial value prior to the onset of a number of tactile or electrical stimulations)—Vm, and the threshold of action potential generation (threshold potential)—Vt. The measurements of the electrical characteristics of the premotor (withdrawal) interneurons were conducted in preparations of snails from all series of experiments.

Since the premotor withdrawal interneurons LPa3 and RPa3 are silent in normal conditions, to generate action potentials in the isolated preparations we applied a depolarizing square-wave form electrical current through the recording microelectrode into the cell for 1 s. For stimulation the minimal current strength for the generation of action potentials was selected; it varied from 1.7 to 3.5 nA.

### Data analyses

The results are shown as mean ± SEM. The unpaired Student's *t*-test and non-parametric Mann–Whitney test were used for comparison between two groups. One-Way ANOVA followed by the Tukey *post-hoc* test and a repeated Two-Way ANOVA were used for comparison between three- or more statistical groups. Independent *t*-tests and the Tukey *post-hoc* test were used to make specific group comparisons. The statistical software SigmaStat32 was used. The statistical significance criterion was *p* < 0.05.

## Results

### Conditioning with neurotoxins

**Figure 1:**
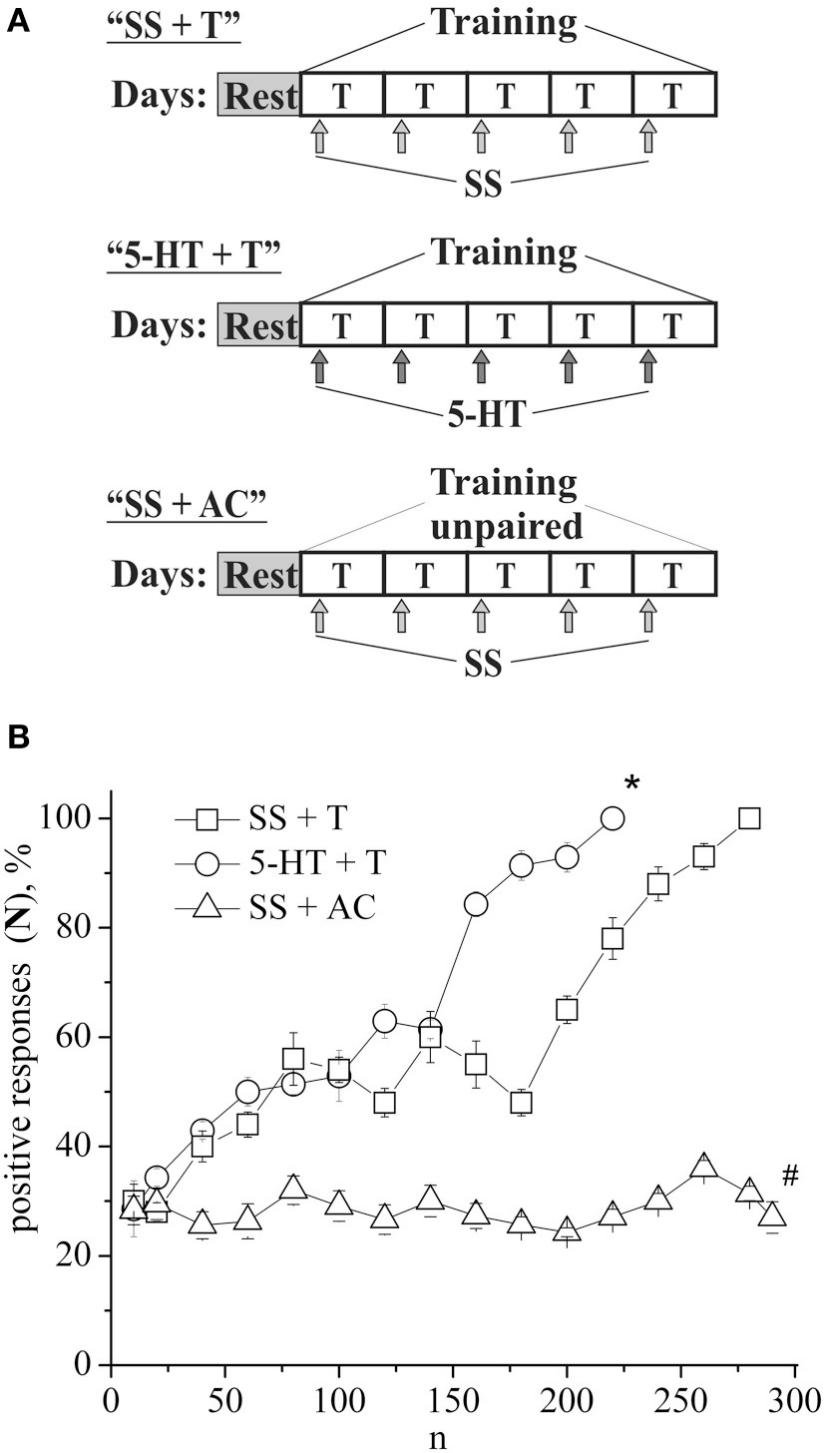
**(A)** Protocol of experiments. **(B)** Dynamics of defensive reflex conditioning on tapping on shell in snails according to the second protocol after daily injections of serotonin (5-HT + T) and saline solution (SS + T) before each training session. N, number of pairings of conditioned and unconditioned stimuli; positive responses (N), %, part of positive responses to conditioned stimulus, as a percentage; 5-HT + T, defensive reflex conditioning during daily injections of serotonin; SS + T, defensive reflex conditioning during daily injections of saline solution; SS + AC, active control—snails received unpaired unconditioned and conditioned stimuli. Vertical axis shows quantity of positive responses of pneumostome (its closure in response to conditioned stimulus), in %; horizontal axis shows numbers of pairs of unconditioned and conditioned stimuli (n). One-Way ANOVA followed by Tukey *post-hoc* test was performed for each time point. Asterisk (^*^) indicates significant difference in the *post-hoc* test between (SS+T)/(5-HT+T) on 220 pairs. Sharp (#) indicate significant differences (SS+T, 5-HT+T) vs. (SS+AC) after the first 30th pairs. (*p* < 0.05, the Tukey *post-hoc* test and independent *t*-test).

**Figure 2:**
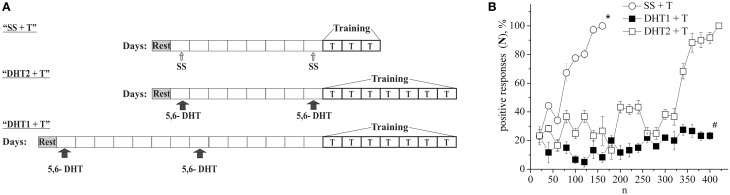
**(A)** Protocol of experiments. **(B)** Dynamics of defensive reflex conditioning on tapping on shell in snails according to the first protocol a week after the second injection of 5.6- DHT (DHT1 + T), the next day after the second injection of the “neurotoxic” analog of serotonin 5.6- DHT (DHT2 + T) and the next day after the saline solution (SS + T) before training session. N, number of pairings of conditioned and unconditioned stimuli; positive responses (N), %, part of positive responses to conditioned stimulus, in percent; DHT1 + T, defensive reflex conditioning a week after the second injection of “neurotoxic” analogs of serotonin 5.6- DHT; DHT2 + T, defensive reflex conditioning the next day after the second injection of “neurotoxic” analogs of serotonin 5.6- DHT; SS + T, defensive reflex conditioning after injection of saline solution. Vertical axis shows quantity of positive responses of pneumostome (its closure in response to conditioned stimulus), in %; horizontal axis shows numbers of pairs of unconditioned and conditioned stimuli (n). Asterisk (^*^) and sharp (#) indicate significant difference (SS+T) vs. (DHT1+T, DHT2+T) and (DHT1+T) vs. (DHT2+T) accordingly. (*p* < 0.05; Two-Way ANOVA and independent *t*-test).

The defensive reflex conditioning on shell tapping was achieved within 6 days with the use of 300–350 repetitions of a combination of shell tapping (conditioned stimulus) and air blowing into pneumostome (unconditioned stimulus) in the case of the second protocol (Figures 1A,B, SS + T) or within 3 days with the use of 150 combinations in the case of first protocol (Figure 2B, SS + T). In both cases the share of positive responses to a conditioned stimulus during training reached 100%. The results of behavioral experiments showed a reliable maintenance of the conditioned defensive reflex for 40 days after training. Snails from the active control group received an identical amount of the conditioned and unconditioned stimuli, but in an unpaired combination. In this case the share of positive responses to a conditioned stimulus in this experimental series during training reached 25–30% (Figure 1B, SS + AC), which proved to be less (*p* < 0.01) than that in the experimental group (up to 100%). It was found that the conditioned defensive reflex wasn't induced in snails (with the first protocol) which were trained the day after the second injection of the “neurotoxic” serotonin analog, 5,6-DHT, (Figure 2B, DHT1 + T). At the same time, in the animals this reflex started to form 1 week after the second injection of the neurotoxin, there was an increased reaction to the conditioned stimulus at the end of the training session and conditioned reflex was successfully elaborated a (Figure 2B, DHT2 + T). From Figure 2A we see that snails begin to learn on 13th day after the second injection of 5,6-DHT. These results probably indicate that 2 weeks after the application of 5,6-DHT the 5-HT system, required for defensive reflex conditioning, begins to recover. It should be noted that the curves for training in naive snails and the snails injected with saline solution did not reliably differ.

### Conditioning with serotonin and 5-hydroxytryptophan

**Figure 3:**
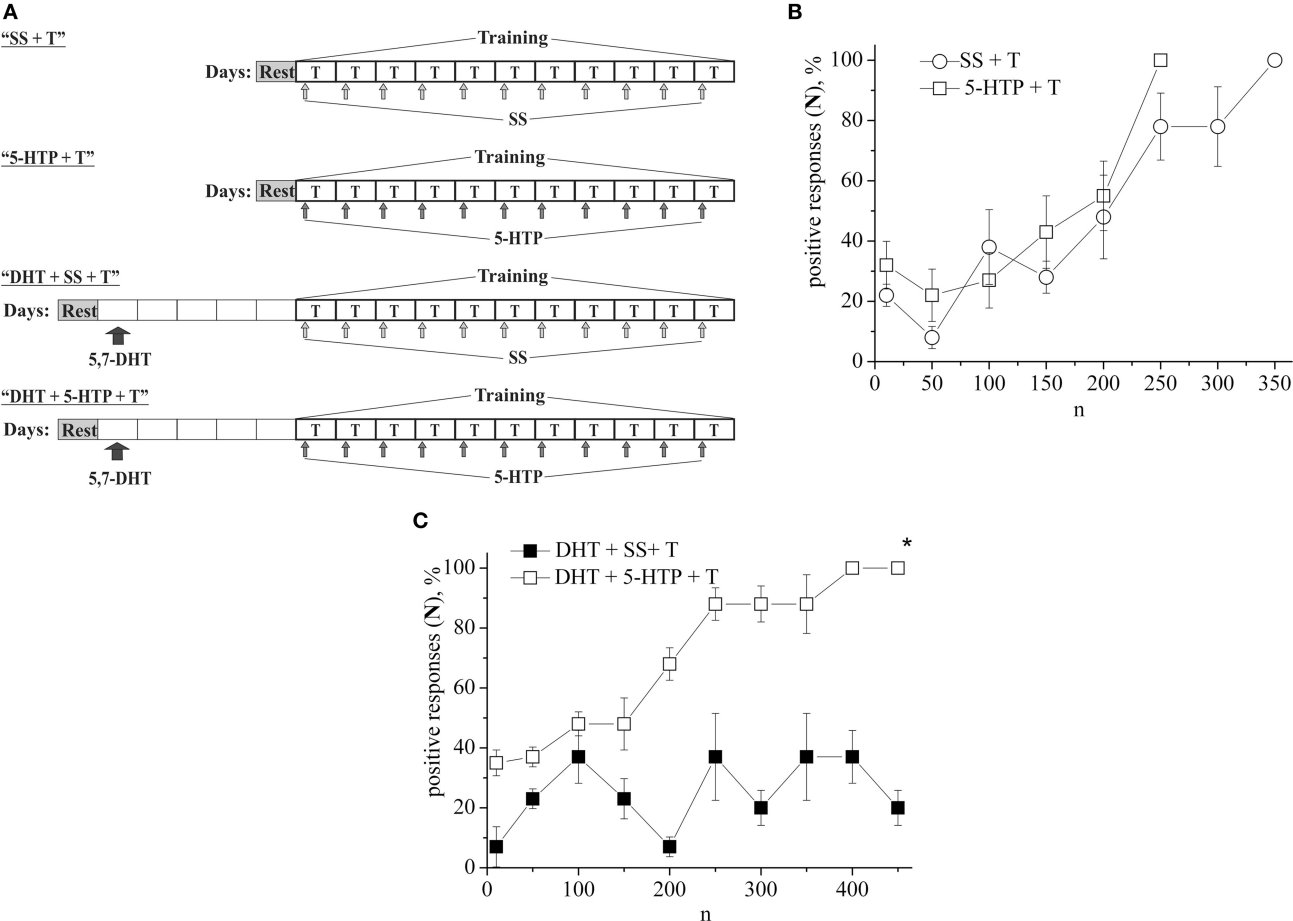
**(A)** Protocol of experiments. **(B)** Dynamics of defensive reflex conditioning on tapping on shell in snails according to the second protocol after daily injection of the precursor of serotonin syntheses 5-HTP before each training session (5-HTP + T) and after daily injection of saline solution (SS + T). **(C)** Dynamics of defensive reflex conditioning on tapping on shell in snails according to the second protocol after a single injection of the “neurotoxic” analog of serotonin 5.7-DHT (DHT + SS + T) and after daily injection of 5-HTP on the background of serotonin deficiency created by the “neurotoxic” analog of serotonin 5.7-DHT (DHT + 5-HTP + T). n, number of pairings of conditioned and unconditioned stimuli; positive responses (N), %, part of positive responses to conditioned stimulus, as a percentage; 5-HTP + T, defensive reflex conditioning after daily injection of 5-HTP before each training session; DHT + T, defensive reflex conditioning after single injection of “neurotoxic” analogs of serotonin 5.7- DHT; DHT + 5-HTP + T, after daily injection of 5-HTP on the background of serotonin deficiency created by the “neurotoxic” analog of serotonin 5.7-DHT; SS + T, defensive reflex conditioning after injection of saline solution. Vertical axis shows quantity of positive responses of pneumostome (its closure in response to conditioned stimulus), in %; horizontal axis shows numbers of pairs of unconditioned and conditioned stimuli (n). Asterisk (^*^) indicates significant difference (DHT+SS+T) vs. (DHT+5-HTP+T) after the first 150th pairs. Two-Way ANOVA revealed a significant DHT effect but no effect (DHT+5-HTP) for disrupt of learning. Interactions were minimal. (*p* < 0.05; Two-Way ANOVA and independent *t*-test).

In the following series of experiments (Figure 3A) the defensive reflex conditioning on shell tapping in snails (the second protocol) was progressed more slowly, so that complete learning was achieved as a result of 350 stimuli combinations (Figure 3B, SS + T). The daily injection of 5-HTP before each training session did not reliably accelerate the defensive reflex conditioning during most of the training (Figure 3B, 5-HTP + T). However, after injections of 5-HTP the snails learned faster. Injection of 5,7-DHT inhibited learning (Figure 3B, DHT + SS + T). From Figure 3A we see that even on the 16-th day after injection of 5,7-DHT snails didn't start to learn. However, daily injection of 5-HTP after the injection of 5,7-DHT restored the snail's ability to learn (Figure 3B, DHT + 5-HTP + T). A daily injection of 5-HT before the training session accelerated the conditioned reflex elaboration (Figure 1B, 5-HT + T).

### Electrophysiological data

**Figure 4:**
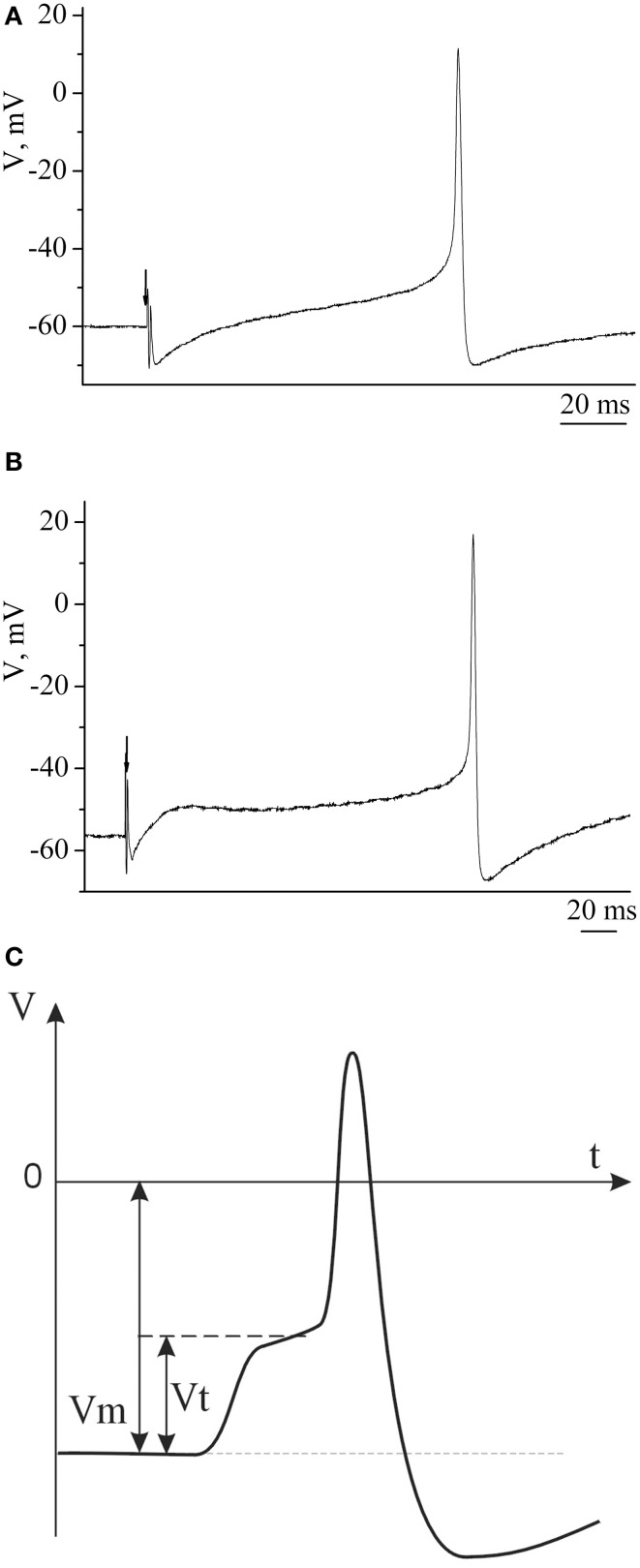
**The action potentials of premotor interneurons of intact (A) and learned (B) snails**. **(C)** Schematic figure of action potential of premotor interneuron and its basic electric characteristics. Vertical axis shows value of potential, in mV; horizontal axis shows time, in ms.

**Figure 5:**
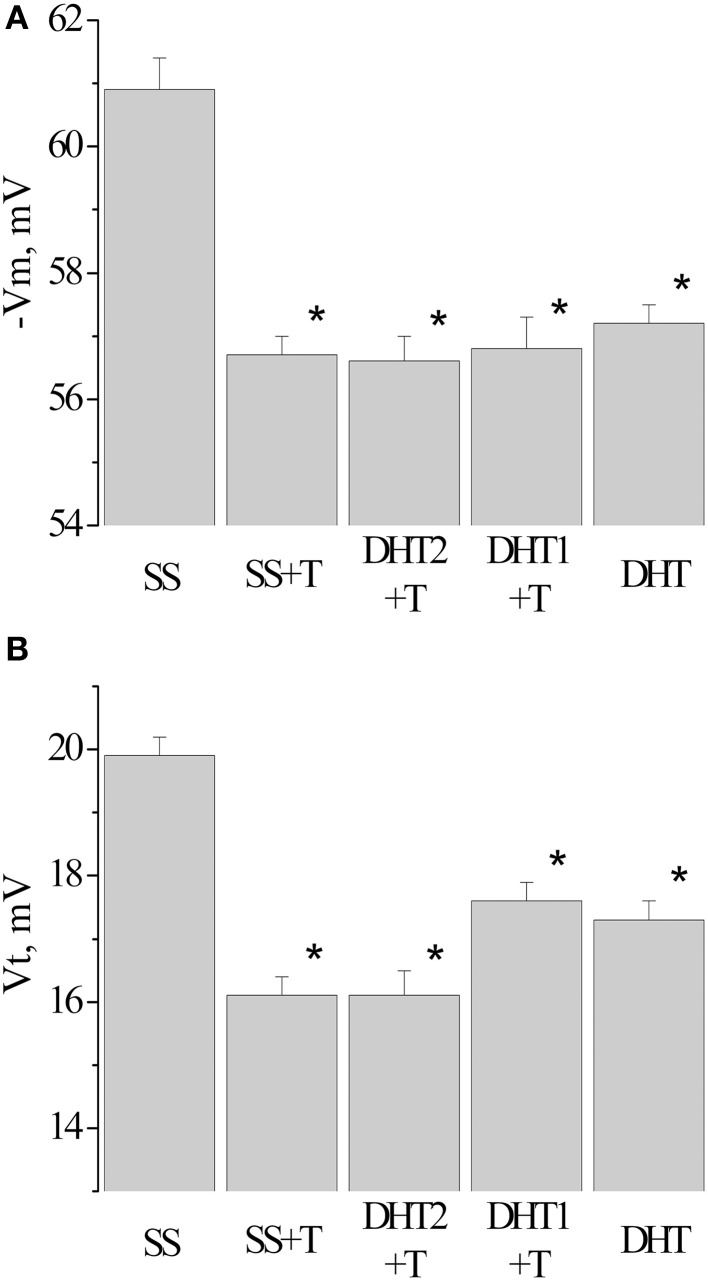
**Value of resting membrane potential—(A) and threshold potential—(B) of premotor interneurons LPa3 and RPa3 in snails in various conditions**. SS, naïve snails; DHT1 + T, defensive reflex conditioning a week after the second injection of the “neurotoxic” analog of serotonin 5.6- DHT; DHT2 + T, defensive reflex conditioning the next day after the second injection of the “neurotoxic” analog of serotonin 5.6- DHT; SS + T, defensive reflex conditioning after injection of saline solution; DHT, snails injected by the “neurotoxic” analog of serotonin 5.6- DHT. ^*^The reliable difference (*p* < 0.001) against active control group (injection of saline solution). Vertical axis shows the value of potential, in mV. ANOVA revealed no T/DHT/5-HT interactions. ^*^p < 0.05; Two-Way ANOVA and independent *t*-test.

Example traces of recorded electrical characteristics of premotor interneurons in naive and trained snails are given in Figures 4A,B. Measurements of electrical characteristics (Figure 4C) showed that the initial membrane resting potential in withdrawal interneurons in the naive snails was −60.9±0.3 mV (*n* = 92), the threshold potential was 19.9 ± 0.4 mV (*n* = 76) (Figures 5A,B—Control). After associative learning (*n* = 74) a reliable decrease in the membrane resting and threshold potentials by 4 mV was observed in the studied interneurons (Figures 5A,B—SS + T). It was found that these changes in the observed electrical characteristics were retained for 1 month. The duration this change in electrical characteristics of premotor interneurons (*n* = 19) has been shown by us earlier (Gainutdinova et al., 2003).

After injection of 5,6/5,7-DHT the depolarization of the membrane in premotor interneurons was observed during recording, both the next day and a week after the injection of 5,6-DHT. The resting membrane potential decreased from −60.3 ± 0.3 mV in SS-injected snails (*n* = 37) to −57.2±0.3 mV in 5,6-DHT-injected snails (*n* = 41), the threshold potential decreased from 19.9 ± 0.3 mV (*n* = 22) to 17.3 ± 0.3 mV (*n* = 33) accordingly (Figures 5A,B—DHT). In snails trained after the second injection of 5,6-DHT no further decrease of the resting membrane and threshold potentials was observed in comparison with the snails injected with 5,6-DHT without training (Figures 5A,B—DHT1 + T, DHT2 + T).

**Figure 6:**
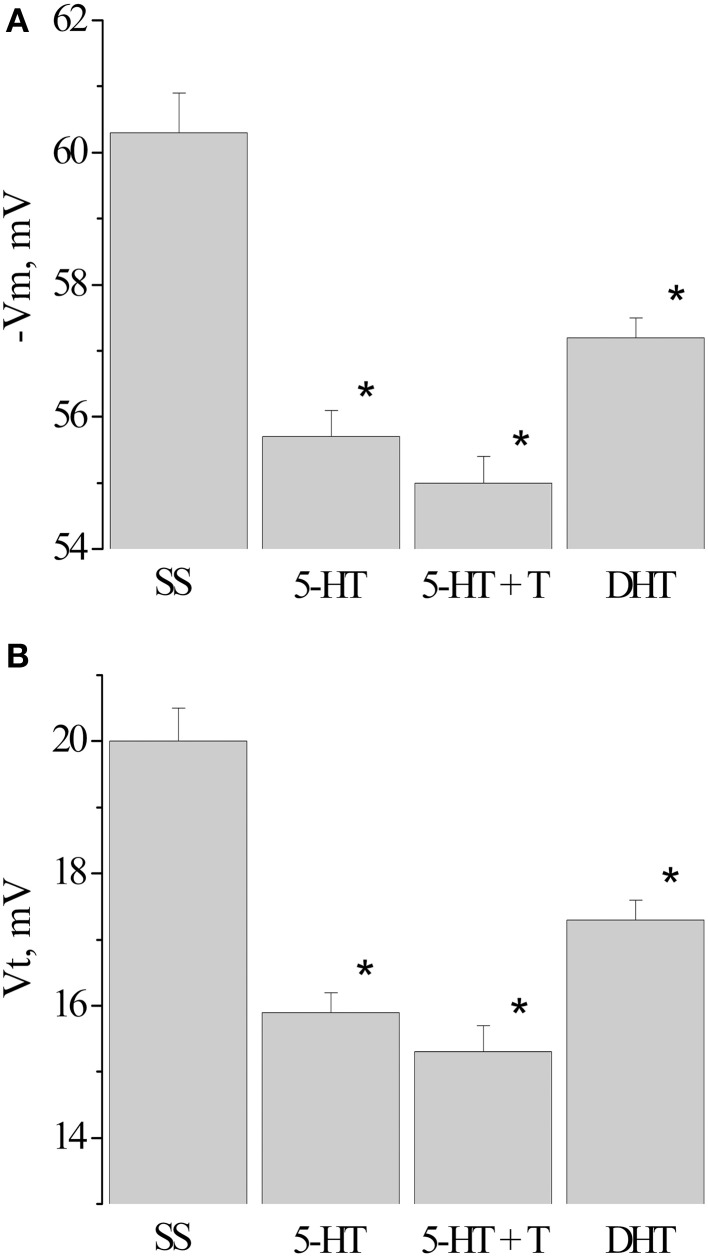
**Value of resting membrane potential—(A) and threshold potential—(B) of premotor interneurons LPa3 and RPa3 in snails learned in various conditions**. SS, naïve snails; 5-HT, snails injected by serotonin; 5-HT+T, defensive reflex conditioning after daily injection of serotonin; DHT, snails injected by the “neurotoxic” analog of serotonin 5.6- DHT. ^*^The reliable difference (*p* < 0.001) against active control group (injection of saline solution). Vertical axis shows the value of potential, in mV. ANOVA revealed no T/DHT/5-HT interactions. ^*^p < 0.05; Two-Way ANOVA and independent *t*-test.

Next, we analyzed the changes in the membrane and threshold potentials of premotor interneurons of snails having received only the injection of 5-HT, and snails, injected with 5-HT before the associative learning. The membrane potential decreased from −60.3 ± 0.6 mV (*n* = 12) to −55.7±0.4 mV (*n* = 13) in snails which received 5-HT only and to −55.0±0.4 mV (*n* = 12) in snails injected with 5-HT before the elaboration of the defensive reflex. In this case in interneurons the value of the threshold potential significantly decreased from 20.0 ± 0.5 mV (*n* = 12) to 15.9 ± 0.3 mV (*n* = 10) and to 15.3 ± 0.3 mV (*n* = 8), respectively (Figure 6, 5-HT, 5-HT + T).

## Discussion

It is well known that learning on the basis of the defensive reflexes of molluscs is mediated by 5-HT (Kandel and Schwartz, 1982; Balaban et al., 2001; Burrell and Sahley, 2005; Gillette, 2006; Il-Han et al., 2010; Hart et al., 2011). The investigations of mechanisms of learning and memory have resulted in new experimental approaches for studying the neurotransmitter and modulator effects of 5-HT, and for studying the mechanisms of participation of corresponding systems in the phenomena of behavioral plasticity (Glanzman et al., 1989; Balaban, 2002, 2008; Barbas et al., 2003; Crow, 2004; Burrell and Sahley, 2005; D'iakonova, 2007; Il-Han et al., 2010; Hart et al., 2011). It is well known that 5-HT induces presynaptic facilitation (Byrne and Kandel, 1996; Lin et al., 2010), it has also been shown that 5-HT can perform integrative functions through its release in the extracellular medium (Sakharov, 1990; Zakharov et al., 1995; Marinesco et al., 2004). These results served as a basis for using 5-HT application to the washing solution as an analog of reinforce stimulus during the formation of cell analogs of learning (Mauelshagen et al., 1996; Liao et al., 1999; Hawkins et al., 2006; Lin et al., 2010; Hu et al., 2011).

### Effects of neurotoxins connected with depletion of serotonin

Our work is devoted to the investigation of mechanisms of associative learning on the basis of the defensive reflex of a terrestrial snail. For the analysis of the role of 5-HT, its temporary deficit using the “neurotoxic” analogs 5,6/5,7-DHT was created. The predecessor for the synthesis of serotonin 5-HTP as well as the injection of 5-HT in the hemolymph of the snail *Helix pomatia* were also used. Our results as in the work of Balaban et al. (1987) show that after depletion of 5-HT the conditioned reflex is not produced. These results correlate with the data of Glanzman et al. (1989) which show the inhibition of the heterosynaptic facilitation in *Aplysia* by the neurotoxin 5,7-DHT. Data from the literature (Pivovarov and Nistratova, 2003; Abramova et al., 2005) and our results show that premotor interneurons respond to 5-HT, i.e., 5-HT may modulate the behavioral effects. Direct evidence of the possibility of electrophysiological modulatory effects of the serotonergic neuron Pd4 on the premotor interneuron LPa3 was found by Balaban et al. (2001).

One of the possible reasons for such effects is a depletion of 5-HT in the nervous system of mollusks by neurotoxins (Gadotti et al., 1986; Glanzman and Krasne, 1986; Jahan-Parwar et al., 1987; Vehosvzky et al., 1989; Kemenes et al., 1990). It was shown early that the “neurotoxic” analogs 5,7-DHT significantly reduced the immunofluorescence staining of 5-HT in the nervous system of crayfish (Glanzman and Krasne, 1986), analysis using HPLC also shows a decrease in the level of 5-HT in the nervous system of *Aplysia* after exposure to this neurotoxin (Glanzman et al., 1989). The results of ultrastructural and biochemical studies showed significant depletion of 5-HT by 5,6-DHT in the first week, and after 21 days the levels of 5-HT returned to normal level (Hernádi et al., 1992; Kemenes, 1997). Our results showed that the snails start to learn on the 13th day after the injection of 5,6-DHT, but after injection of 5,7-DHT snails hadn't even started to learn on the 16-th day.

### Excitability of premotor interneurons and learning

Earlier we found that the defensive reflex conditioning in snails is accompanied by a depolarization shift of the membrane potential and a decrease of the threshold potential (Gainutdinov et al., 1998). In recent years there have been a sufficient number of experimental results that demonstrate the membrane correlates of learning (Gillette et al., 1982; Alkon, 1984; Frysztak and Crow, 1997; Cleary et al., 1998; Gainutdinova et al., 2003; Disterhoft and Oh, 2006; Kemenes et al., 2006; Nikitin et al., 2006, 2013; Mozzachiodi et al., 2008; Jing et al., 2009; Debanne and Poo, 2010; Gainutdinov et al., 2011; Sakharov, 2012; Cavallo et al., 2014a,b). These experiments were done on preparations from trained animals as well as within the cell analogs of learning.

A number of questions arise during the analysis of the effects of 5-HT. It is known that one of the main functions of 5-HT both in vertebrates and invertebrates is to facilitate the motor output. For example, the facilitation action on reflex activity and on central pattern generators (Gillette, 2006). The increase in excitability of neurons under the action of 5-HT has been noted by a number of authors (Frysztak and Crow, 1997; Liao et al., 1999; Balaban et al., 2001; Pivovarov and Nistratova, 2003; Abramova et al., 2005; Dumitriu et al., 2006; Hawkins et al., 2006). Jin and co-authors have shown that 5-HT increases the peak amplitude of the complex excitatory post-synaptic potential induced by light, and also increases the internal excitability and the spike activity of type Ie(A) interneurons of the mollusk *Hermissenda* (Jin et al., 2009). In contrast 5-HT reduces the spike activity and internal excitability of type Ie(B) interneurons. We found two effects in our experiments: the depolarization shift of membrane potential and decrease in the threshold potential of premotor interneurons after training and after the injection of the 5-HT. There is an absence of a summing effect of these two factors. The depolarization shift of membrane potential and decrease in threshold potential of premotor interneurons in response to injection of 5-HT and its “neurotoxic” analogs to intact snails is a possible consequence of coupling of these substances (5-HT and neurotoxins) with 5-HT receptors.

### Serotonin receptors in premotor interneurons

In our experiments we have shown that injections of the neurotoxins 5,6/5,7-DHT is accompanied by the depolarization of premotor interneurons and a decrease in their threshold potential, as with injections of 5-HT. The question arises, are the depolarization shift of membrane potential and decrease in threshold potential of premotor interneurons after injection of 5-HT and its neurotoxic analogs the result of their interaction with 5-HT- receptors due to the structural similarity between 5-HT and its neurotoxic analogs? These common effects suggest that they are related to the effects of 5-HT on receptors located on the membrane of premotor interneurons and possibly on the intermediate neurons that are presynaptic to premotor interneurons. Pivovarov and Nistratova (2003) analyzed the possible presence of 5-HT receptors on the soma of snail's premotor interneurons. 5-HT, applied locally to the soma, reversibly decreased the input current caused by acetylcholine (local ionophoretical application). They demonstrated that only NAN-190 (a 5-HT_1A_-receptor antagonist) and methiothepin (a 5-HT_1E_ receptor antagonist) inhibited the development of the 5-HT effect. The results show the presence of 5-HT receptors but only of the first type on the soma of *Helix* premotor interneurons (Pivovarov and Nistratova, 2003; Abramova et al., 2005).

It is known that the 5-HT1A receptor is among the most abundant and widely distributed 5-HT receptors in the brain, but is also expressed on 5-HT neurons as an autoreceptor where it plays a critical role in regulating the activity of the entire 5-HT system and over-expression of the 5-HT1A autoreceptor has been implicated in reducing 5-HT neurotransmission (Albert et al., 2011). Our results showed that 5-HT and its neurotoxin produced similar effects. It is possible to consider the results obtained with 5-HT1A autoreceptors. It has been shown that most if not all 5-HT1A autoreceptors on the plasma membrane of soma-dendrites from nucleus raphe dorsalis are located extrasynaptically (Riad et al., 2004). Therefore, 5-HT and its neurotoxin may bound with these autoreceptors and decrease the effect of 5-HT. However, the question remains whether the 5-HT1A receptors in the premotor interneurons are autoreceptors?

It has been shown that the broadening of the action potential of *Aplysia* sensory neurons in response to 5-HT application is mediated by 5-HT receptors of the first type, blocked by methiothepin (Dumitriu et al., 2006). K. Lukowiak et al studied the role of the 5-HT- system in the responses of the mollusk *Lymnaea* to the danger stimulus. Using mianserin, a 5-HT receptor antagonist they found the disruption of two types of defensive behavior (increase in the time of exit of freshwater snail from their shell and the shadow reflex), caused by an extract of crab tissue (Il-Han et al., 2010). Methysergide, another 5-HT receptor antagonist had the same effect, blocking the formation of long-term memory after training with an extract of crab tissue. However, importantly, mianserin didn't affect formation of long-term memory after training in water without an extract of crab tissue. These data suggest that the 5-HT- system is activated only with danger detection. These results show the possibility of an extracellular action of 5-HT. The differences in the responses to 5-HT led to the opinion that there are different subtypes of 5-HT receptors in the nervous system of *Aplysia* (Barbas et al., 2003). The possibility of participation of different types of 5-HT receptors in different signaling pathways has also been demonstrated in the work of Kiss et al. (2003). Since potentiation of S-cells in the leech was blocked by the 5-HT receptor antagonist methylsergide, it was concluded that this metabotropic receptor is involved in the regulation of excitability of S-cells (Burrell and Sahley, 2005). Other researchers have cloned a 5-HT receptor called 5-HT(apAC1), which stimulates the production of cAMP, the inhibition of which blocks synaptic facilitation of the sensorimotor synapse of *Aplysia* (Lee et al., 2009). A 5-HT receptor, which regulates protein kinase C PKC has also been found, called Apl II (Nagakura et al., 2010).

### Effects of serotonin and 5-hydroxytryptophan injections on learning

It is known that 5-HT is an important mediator of defensive behavior in molluscs (Whitaker-Azmitia, 1999; Gillette, 2006; D'iakonova, 2007; Hernadi et al., 2008; Lee et al., 2009). It has been shown that the 5-HT transmission from modulatory neurons to premotor interneurons includes the release of 5-HT from modulatory neurons into the extracellular medium (Zakharov et al., 1995; Balaban et al., 2001). We found that daily injection of 5-HT accelerated the defensive reflex conditioning in snails. This result is similar to those found by Lee et al. (2008). They found that one pulse of 5-HT produces a transient facilitation mediated by the cAMP-dependent protein kinase leading to covalent modifications in the sensory neurons which results in an enhancement of transmitter release and a strengthening of synaptic connections lasting minutes. By contrast, repeated pulses of 5-HT induce a transcription- and translation-dependent long-term facilitation lasting more than 24 h and trigger the activation of a family of transcription factors in the presynaptic sensory neurons including ApCREB1, ApCREB2, and ApC/EBP. Other researchers have also shown that 5-HT-induced long-term facilitation of the Aplysia sensorimotor synapse depends on enhanced gene expression and protein synthesis (Villareal et al., 2007; Hart et al., 2011). There is evidence that one of these proteins could be synapsin (Fioravante et al., 2007; Hart et al., 2011).

5-HTP didn't reliably accelerate the defensive reflex conditioning during training, however, after injection of 5-HTP learning in general was achieved faster. Our results demonstrate that daily injection of 5-HTP before a training session in snails with a 5-HT deficiency, created by the “neurotoxic” analog of serotonin 5,7-DHT, restored the ability of snails to learn. The explanation for this fact can be found in the data of Fickbohm et al. (2005). Using high performance liquid chromatography and immunochemistry they showed a significant increase in 5-HTP content for over 20 h in the brain of a mollusk *Tritonia* after 30 min standing in a solution of 2 mM 5-HTP and they also showed the increase of 5-HT in specific areas of the brain (Fickbohm et al., 2005). The difference in our experiments is that we not only injected snails with 5-HT and 5-HTP but also elaborated a conditioned reflex, i.e., had to deal with the simultaneous action of two factors. So the question arose by what mechanisms does 5-HT accelerate learning? It is known that learning is the result of changes in presynaptic processes, such as direct modulation of the release of neurotransmitters and post-synaptic processes, such as the properties of receptors (Kandel, 1976, 2001; Hawkins et al., 2006; Balaban, 2008; Mozzachiodi and Byrne, 2010; Vavoulis et al., 2010; Balaban et al., 2014). At the same time the effect of recovery in the ability to learn caused by injections of 5-HTP on the background of deficiency of 5-HT created by 5,7-DHT, demonstrates a partial maintenance of the functioning of the 5-HT synapses.

## Conclusion

In conclusion, we have shown that a daily injection of 5-HT before a training session accelerated learning, and daily injection of 5-HTP before a training session in snails with a 5-HT deficiency (caused by the “neurotoxic” analogs of serotonin 5,7-DHT), restored the ability of snails to learn. The results suggest that during learning 5-HT is released into the extracellular medium, which interacts with receptors located on the membrane of premotor interneurons and possibly on the intermediate neurons that are presynaptic to premotor interneurons. Learning is also accompanied by a decrease in membrane and threshold potentials of premotor interneurons.

### Conflict of interest statement

The authors declare that the research was conducted in the absence of any commercial or financial relationships that could be construed as a potential conflict of interest.

## Abbreviations

5, 6- and 5, 7 DHT: 5, 6- and 5, 7- dihydroxytryptamine
5-HTP: 5-hydroxytryptophan
SS: saline solution
5-HT: serotonin.
